# Kynurenine Metabolites in CSF and Plasma in Healthy Males

**DOI:** 10.1177/11786469241245323

**Published:** 2024-04-24

**Authors:** Funda Orhan, Lilly Schwieler, Göran Engberg, Martin Samuelsson

**Affiliations:** 1 Department of Physiology and Pharmacology, Karolinska Institutet, Stockholm, Sweden; 2 Center for Social and Affective Neuroscience, Department of Biomedical and Clinical Sciences, Linköping University, Sweden; 3 Department of Psychiatry, Linköping University Hospital, Sweden

**Keywords:** Tryptophan, kynurenine, kynurenic acid, quinolinic acid, CSF, plasma, urine

## Abstract

In recent years, kynurenine metabolites generated by tryptophan catabolism have gained increasing attention in the context of brain diseases. The question of importance is whether there is a relationship between peripheral and central levels of these metabolites. Some of these compounds do not cross the blood-brain barrier; in particular, kynurenic acid, and most analyses of kynurenines from psychiatric patients have been performed using plasma samples. In the present study, we recruited 30 healthy volunteers with no history of psychiatric or neurological diagnosis, to analyze tryptophan, kynurenine, kynurenic acid, and quinolinic acid levels in CSF and plasma. In addition, kynurenic acid was analyzed in urine. The most important finding of this study is that CSF kynurenic acid levels do not correlate with those in plasma or urine. However, we found a correlation between plasma kynurenine and CSF kynurenic acid. Further, plasma kynurenine and plasma quinolinic acid were correlated. Our findings clarify the distribution of tryptophan and its metabolites in various body compartments and may serve as a guide for the analysis of these metabolites in humans. The most significant finding of the present study is that a prediction of brain kynurenic acid by of the analysis of the compound in plasma cannot be made.

## Introduction

**Figure 1.**
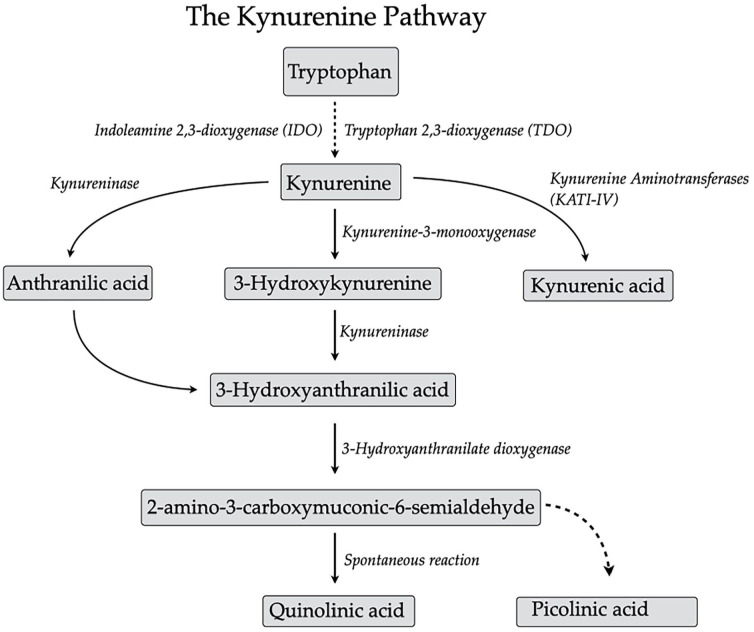
The kynurenine pathway of tryptophan degradation.

Tryptophan (TRP) is an essential amino acid that is primarily degraded via the kynurenine (KYN) pathway (KP; Figure 1).^1^ This process generates several physiologically active compounds that are linked to diverse conditions like psychiatric, cardiovascular, neurodegenerative, immunological, infectious disorders, and cancer.^2-10^

The enzymatic degradation of TRP along the kynurenine pathway occurs throughout the body, and pathway enzymes are found in many organs/cell types, including the liver, kidneys, brain, and immune cells. The rate-limiting enzymes indoleamine 2,3-dioxygenase (IDO)1, IDO2, and tryptophan dioxygenase 2 (TDO2) are responsible for opening the indole ring of TRP, resulting in the formation of formylkynurenine. Kynurenine formylase then converts formylkynurenine to L-KYN, the key compound of the kynurenine pathway. Peripherally produced KYN easily cross the blood-brain barrier via the large neutral amino acid carrier (L-system),^11^ and as much as 60% to 80% of brain KYN is derived from peripheral tissues.^12,13^ Degradation of brain KYN occurs in astrocytes and microglia, producing the neuroprotective kynurenic acid (KYNA) and the neurotoxic metabolite quinolinic acid (QUIN), respectively. 3-HK and anthranilic acid (ANTRA) are intermediate products of the kynurenine pathway and contribute to the synthesis of other neuroactive metabolites such as QUIN and picolinic acid (PIC), However, KYNA, QUIN, and PIC cross the blood-brain barrier poorly.^14^ Brain KYNA is an end-metabolite and its production is highly dependent on the availability of KYN^15^ and involves an irreversible transamination process catalyzed by kynurenine aminotransferases (KATs). KAT enzymes have been identified as 4 distinct homologs (KAT I-IV).^16-20^ KAT II stands out as the main KYNA biosynthetic enzyme under physiological conditions in the mammalian brain.^21-23^ and accounts for approximately 70% of KYNA production in the human brain^20^ and 75% in the rat brain under physiological conditions.^22^ QUIN and PIC are preferably produced in microglia, a neurotoxic branch of the kynurenine pathway, by kynurenine monooxygenase (KMO) and kynureninase (KYNU).^24^

The synthesis of kynurenine predominantly take place in immune-related cells like microglia and astrocytes, suggesting a significant link between the immune system and the activity of the kynurenine pathway. In particular, proinflammatory cytokines stimulate this pathway, resulting in increased synthesis of KYN that will lead to increased production of all physiologically active compounds of the pathway^8,25-27^ KYNA, the most extensively studied metabolite, is an antagonist of glutamate receptors in the human brain, and at low concentrations, the compound blocks the glycine site of the N-methyl-D-aspartate (NMDA) receptor (IC50 = 8-15 µM) as well as the α7 nicotinic acetylcholine receptors (IC50 = 7 µM).^28-31^ At higher concentrations, the glutamate recognition site of NMDA receptors (IC50 = 200-500 µM)^29^ and alpha-amino-3-hydroxy-5-methylisoxazole propionate (AMPA)/kainate receptors are blocked (IC50 in the millimolar range).^29,32^ Furthermore, KYNA has been shown to be an endogenous agonist of the G protein-coupled receptor 35 (GPR35; EC50 = 39 µM in humans)^33^ and the aryl hydrocarbon receptor.^34^ Under normal conditions, tryptophan metabolites are eliminated from the brain via probenecid-sensitive transporters that adjust the levels of KYNA and other TRP metabolites in various compartments.^35,36^ Rapid renal excretion seems to constitute the single most prominent mechanism of brain KYNA disposition.^37^ On the other hand, QUIN is an NMDA receptor agonist, acting through NMDA receptors containing the NR1 + NR2A and the NR1 + NR2B subunits.^38^ In addition, QUIN decreases glutamate uptake and recycling by astrocytes and increases neuronal glutamate release. Hence QUIN is a potent excitotoxin also with proinflammatory and immunoregulatory properties.^39^

Given the proposed role of kynurenines in a variety of neurological and psychiatric diseases, numerous studies have analyzed these compounds in the blood. However, relying solely on blood analysis may not accurately predict brain tryptophan levels and its metabolites. The primary objective of the present study was to quantify levels TRP and its metabolites in CSF and blood of healthy males. Another goal was to establish correlations between peripheral kynurenine levels and those found in the CSF, tentatively allowing a prediction of the concentration of CSF kynurenines based on more accessible peripheral measurements.

## Material and Methods

### Subject population

Thirty healthy volunteers were recruited among medical students, hospital staff members, and relatives. Male subjects were only included in this study since females undergoes an estrus cycle that appears to affect the concentrations of TRP metabolites, thereby complicating result interpretation.^40^ All participants underwent a medical check-up, including laboratory tests (electrolytes, blood, kidney, liver, and thyroid) and a physical examination including weight, height, and back length which appears negatively correlated to a wide variety of CSF metabolites in general, including KYNA^41^ (Table 1). The volunteers had to have been medication-free for at least 1 month and free from any form of substance abuse. Smoking was allowed.

**Table 1.** Demographic characteristics of the male healthy volunteers.

Characteristics	Mean ± SD (n) [range]
Age (y)	25.3 ± 7.2 (30) [18-51]
Height (cm)	182.2 ± 7.5 (30) [172-200]
Weight (kg)	79.3 ± 9.8 (30) [58-108]
BMI (kg/m^2^)	23.9 ± 2.9 (30) [19-33]
Back length (cm)	64.0 ± 4.8 (28) [56-73]

Abbreviation: BMI, body mass index.

The volunteers were subjected to a semi-structured interview using the Structured Clinical Interview of DSM-IV Axis I Disorders (SCID-I). The interviews were directed towards a history of affective disorders, anxiety disorders, and drug abuse. The volunteers also completed the SCID-II questionnaire for personality disorders.

### Collection of samples

Blood samples were drawn at 8.00 am, 12 noon, 4.00 pm, and 8.00 pm (Day 1, no restriction of food intake). On the following day (Day 2), at 8.00 am in a fastening state, one blood sample was drawn, and the lumbar puncture was performed. All urine was collected within 24 hours before lumbar puncture was performed.

### Plasma

All blood samples were centrifuged in a Sigma 203 centrifuge at 3500 rpm (1438×*g*) for 10 minutes. Plasma was separated immediately and frozen at −70°C until analysis.

### Urine

10 ml of the 24-hour urine was collected and frozen at −70°C until analysis. Urine was analyzed for creatinine.

### Lumbar puncture

Lumbar puncture was performed after a minimum of 8 hours of fasting. There were no restrictions concerning posture or rest during the preceding 8 hours. At approximately 8 am, a disposable needle (BD Whitacre Needle 0.7 × 90 mm) was inserted at the L 4-5 level with the subject in the right decubitus position. For convenience, a pillow was placed under the subject’s head. CSF was allowed to drip into a plastic test tube. Since a rostrocaudal gradient is often observed when analyzing CSF biomarkers, three 6-ml fractions were sampled (0-6, 7-12, and 13-18 ml), protected from light, and centrifuged in a Sigma 203 at 3500 rpm (1438*g*) for 10 minutes within 30 minutes after the puncture. Each 6-ml sample was divided into three 2-ml aliquots, placed in a freezer (−70°C) until analysis.

### Analysis of kynurenic acid

The analysis of KYNA was performed utilizing an isocratic reversed-phase high-performance liquid chromatography (HPLC) system, including a dual-piston, high liquid delivery pump (Bischoff, Leonberg, Germany), a ReproSil-Pur C18 column (4 × 150 mm, Dr. Maisch GmbH, Ammerbuch, Germany), and a fluorescence detector (Jasco Ltd., Hachioji City, Japan) with an excitation wavelength of 344 nm and an emission wavelength of 398 nm (18 nm bandwidth). A mobile phase of 50 mM sodium acetate (pH 6.2, adjusted with acetic acid) and 7.0% acetonitrile was pumped through the reversed-phase column at a flow rate of 0.5 ml/min. Samples of 50 µl were manually injected into the system (ECOM, Prague, Czech Republic). Zinc acetate, 0.5 M (not pH adjusted), was delivered post column by a peristaltic pump (P-500, Pharmacia, Uppsala, Sweden) at a flow rate of 0.10 ml/min. Signals from the fluorescence detector were transferred to a computer for analysis with Datalys Azur (Grenoble, France). The retention time of KYNA was approximately 7 to 8 minutes. Initially, the sensitivity of the system was verified by analysis of a standard mixture of KYNA with concentrations ranging from 0.5 to 30 nM resulting in a standard linear plot. To verify the reliability of this method, some samples were analyzed in duplicate, and the mean intra-individual variation was below 5%.

### Analysis of tryptophan, kynurenine, and quinolinic acid

Detection was performed using a Waters Xevo TQ-S triple quadrupole mass spectrometer operating in a positive-ionization MS/MS configuration. The mobile phase was run at a flow rate of 300 μl/min and consisted of 2.1% formic acid in MilliQ water (A phase) and 95% acetonitrile 0.1% formic acid (B phase), starting with 5% B for 2 minutes, followed by gradient elution, with a total run time of 10 minutes. The mass spectrometer was tuned for QUIN, KYN, TRP, and KYNA, and the mass spectral transitions were set at m/z 168 > 106, 209 > 146, 205 > 118, and 190 > 116 and for the internal standards 172 > 110 (13C315N1-QUIN), 213 > 150 (D4-KYN), 210 > 123 (D5-TRP), and 194 > 120 (D5-KYNA). QUIN, TRP, D5-TRP, KYN, and KYNA were purchased from Sigma–Aldrich, 13C315N1-QUIN from Synfine Research Inc., D4-KYN and D5-KYNA from Buchem B.V. Calibration was performed using standards that covered the CSF concentration range. Lowest levels of detection for TRP, KYN, and QA were 100, 10, and 5 µM, respectively. Seven concentration points were used to establish a linear calibration curve for quantification. The calibration curve was plotted using the ratio of the analyte peak area to the IS peak area after integration using Masslynx 4.1 software (Waters Corporation). The retention times for QUIN, KYN, TRP, and KYNA were 1.2, 1.7, 3.6, and 3.9 minutes respectively.

### Statistics

All statistical analysis was performed using Statistica 8 software (StatSoft, Tulsa, OK, USA) and SPSS statistics version 28 (IBM, Armonk, NY, USA). Continuous variables were analyzed by Pearson’s correlation analysis (or partial correlation analysis) as indicated. Correlations were made between body measures (age, height, weight, BMI, and back length), urine-KYNA/plasma-creatinine, CSF TRP metabolites, and plasma TRP metabolites. Comparisons of TRP metabolites within each time point were performed using ANOVA with Bonferroni correction. For group comparisons, student’s *t*-test for independent variables was used to compare groups, whereas student’s *t*-test was used when comparing dependent samples. A *P*-value <.05 was considered to be significant. A power test (https://sample-size.net/correlation-sample-size/) was employed to determine the requisite sample size for assessing correlation. The analysis indicated that a minimum of 29 samples is necessary to achieve statistical power with a significance level of *P* < .05.

### Policy and ethics

The study adhered to principles embodied in the Declaration of Helsinki.^42^ All enrolled healthy males consented orally and in writing to participate in the study. The study was approved by the Ethical Committees of the University Hospital in Linköping, Sweden.

## Results

Demographic characteristics of participants are presented in Table 1. The concentrations of TRP, KYN, QUIN, and KYNA in CSF and plasma are displayed in Table 2.

**Table 2.** Tryptophan metabolites in plasma and CSF.

Samples	Mean ± SD (n)			
	KYNA (nM)	TRP (µM)	KYN (µM)	QUIN (nM)
*Plasma*				
Day 1: Time: 8.00 am	40.95 ± 10.08 (30)			
Day 1: Time: 12.00 noon	40.71 ± 9.64 (30)			
Day 1: Time: 4.00 pm	40.10 ± 13.11 (30)			
Day 1: Time: 8.00 pm	40.14 ± 11.01 (30)			
Day 2: Time: 8.00 am	39.95 ± 9.82 (29)	73.98 ± 11.95 (18)	2.06 ± 0.44 (29)	608.0 ± 196.4 (18)
Mean (day 1 and 2)	40.31 ± 8.63			
*CSF*				
Fraction: 0-6 ml	1.36 ± 0.61 (29)	2.06 ± 0.29 (28)	0.05 ± 0.02 (23)	
Fraction: 7-12 ml	1.41 ± 0.50 (29)	1.80 ± 0.32 (30)	0.03 ± 0.01 (30)	18.02 ± 6.12 (18)
Fraction: 13-18 ml	1.47 ± 0.45 (29)	1.80 ± 0.32 (30)	0.03 ± 0.01 (30)	
Fraction: 1-12 ml	1.39 ± 0.53 (29)	1.94 ± 0.19 (28)	0.04 ± 0.01 (23)	
Mean: 18 ml	1.42 ± 0.49 (29)	1.89 ± 0.17 (28)	0.04 ± 0.01 (23)	

Abbreviations: CSF, cerebrospinal fluid; KYN, kynurenine; KYNA, kynurenic acid; QUIN, quinolinic acid; TRP, tryptophan.

### Tryptophan

In CSF, there was an overall difference in the 3 TRP fractions (*P* < .01) with higher values in fraction 0 to 6 ml (2.06 ± 0.29) than in 7 to 12 ml (1.82 ± 0.32) and 13 to 18 ml (1.81 ± 0.32), although no differences between fraction 7 to 12 ml and 13 to 18 ml was observed. Plasma TRP correlated with weight (*r* = .61, *P* < .01) and BMI (*r* = .65, *P* < .01) as well as with plasma QUIN (*r* = .64, *P* < .01). No correlations were found between plasma TRP or any TRP metabolite in CSF. Coffee consumption was related with higher concentrations of CSF TRP in fraction 0 to 6 ml (2.16 ± 0.25 µmol/l, n = 20) than those not drinking coffee (1.77 ± 0.30 µmol/l, n = 5) (*P* < .01). Smoking did not affect plasma or CSF TRP.

### Kynurenine

In CSF, there was an overall difference in the 3 KYN fractions (*P* < .001) with higher values in fraction 0 to 6 ml (0.05 ± 0.02) than fractions 7 to 12 ml (0.03 ± 0.01) or 13 to 18 ml (0.03 ± 0.01), but no differences were observed between fractions 7 to 12 ml and 13 to 18 ml. We observed no correlation with body measures or age. Correlations were found between plasma KYN and all CSF KYNA fractions (*P* = .004–.019, Table 3) as well as with plasma QUIN (*r* = .51, *P* < .05). When all fractions were considered (0-18 ml) CSF KYN correlated with plasma QUIN (*r* −.66, *P* < .05). There was a tendency for correlation between plasma KYN and plasma KYNA. No correlations between KYN in plasma and CSF were established. Smoking or coffee consumption did not affect KYN levels in plasma or CSF.

**Table 3.** Correlations between plasma KYN and different fractions of CSF KYNA.

CSF KYNA^a^	*r*-Value^b^	*r*-Value (adjusted)^c^	*P*-value^b^	*P*-value (adjusted)^c^
Fraction: 0-6 ml	.44	.46	.019	.015
Fraction: 7-12 ml	.47	.47	.013	.014
Fraction: 13-18 ml	.52	.53	.004	.004
Fraction: 1-12 ml	.47	.49	.011	.010
Fraction: 0-18	.50	.52	.007	.006

Abbreviations: CSF, cerebrospinal fluid; KYN, kynurenine; KYNA, kynurenic acid.

a Samples were taken simultaneously at 08.00 am.

b Pearson’s correlation.

c Pearson’s partial correlation, *P*-value, and *r*-value adjusted for creatinine.

### Kynurenic acid

KYNA concentration did not differ between the 3 CSF fractions. Further, plasma KYNA concentration appeared very stable since no overall differences in plasma KYNA concentrations at various time points during Day 1 and 2 were found. Neither fasting nor breakfast affected the plasma concentration of KYNA since the 8 am plasma sample did not differ between Day 1 and Day 2. Body height correlated positively with plasma KYNA (*r* = .39, *P* < .05) and with urine KYNA/creatinine ratio (*r* = .38, *P* < .05), but no other correlations with body measures or age were found. Urine KYNA/creatinine correlated with all plasma KYNA values (mean plasma *r* = .61, *P* < .001). Notably, neither fraction of CSF KYNA correlated with plasma levels of KYNA, but all fractions of CSF KYNA correlated with plasma KYN levels (Table 3). Plasma KYNA at 4.00 p.m. correlated with CSF KYN when fractions were considered, that is, 0 to 18 ml (*r* .46, *P* < .05). When sampled simultaneously on Day 2 plasma KYNA correlated with plasma KYN (*r* = .42 *P* = .02) and tended to correlate with plasma QUIN (*r* = .41, *P* = .09). Smokers, compared to non-smokers, had lower concentrations of KYNA in CSF fraction, 7 to 12 ml (smoker 0.84 ± 0.20 n = 3, non-smoker 1.47 ± 0.48 n = 24, *P* < .05), fraction 13-18 ml (smoker 0.87 ± 0.08 n = 3, non-smoker 1.55 ± 0.42 n = 24, *P* < .01), or when all fractions were considered 1 to 18 ml (smoker 0.85 ± 0.17 n = 3, non-smoker 1.48 ± 0.47 n = 24, *P* < .05). Smoking did not affect urine KYNA levels. Coffee consumption did not affect KYNA in plasma, CSF, or urine.

### Quinolinic acid

In contrast to CSF QUIN, plasma QUIN correlated with plasma TRP, CSF KYN when all fractions were considered, 0 to 18 ml, and plasma KYN. Neither plasma QUIN, nor CSF QUIN correlated with body measures or age. Smoking or coffee consumption did not affect QUIN in plasma or CSF.

## Discussion

Several studies have shown the involvement of KYNA and QUIN in mental disorders; however, most studies have been performed using plasma, and neither KYNA nor QUIN passes the blood-brain barrier. The rising interest in KYNA and QUIN in several mental disorders makes it essential to further explore the distribution of KYNA in various body fluids and their inter-relationships. The most important finding of the present study is that CSF KYNA levels did not correlate with those in the plasma.

A diurnal rhythm of KYNA production, with higher daytime production, has been suggested in humans.^43^ In the present study, limited to only healthy males, no variations in KYNA levels in plasma were observed during the daytime. Fasting during the night did not influence KYNA levels in morning samples. Thus, at least in healthy males, it is possible to obtain a representative estimation of plasma KYNA, regardless of sampling at different times of the day. Furthermore, the correlation between urine and plasma KYNA makes it feasible to estimate daytime plasma KYNA from a 24-hour urine KYNA sample. However, previous reports on KYNA’s diurnal rhythm in plasma and urine have shown that there is a need for further studies regarding the correlation between urine KYNA and plasma KYNA levels at different times of the day.^43^ Furthermore, there was no correlation between plasma and CSF KYNA levels. Nevertheless, the correlation between fastening levels of plasma KYN and CSF KYNA may open up new avenues for the prediction of CSF KYNA from a blood sample. This is in line with the notion that KYN, the precursor of KYNA, passes the BBB, whereas KYNA does not due to its polar structure. Therefore, it is believed that KYNA detected in the CSF is most likely of central origin. Notably, though, there was no correlation between plasma KYN and CSF QUIN levels, or between concentrations of KYN in plasma and CSF.

When analyzing various biomarkers in CSF using lumbar puncture, a rostrocaudal gradient is often observed.^44,45^ However, we found no gradient in the CSF for KYNA. In contrast, gradients for CSF KYN and CSF TRP were found with higher concentrations in fraction 0 to 6 ml than in fraction 7 to 12 ml and 13 to 18 ml. When performing a lumbar puncture (LP), a small-volume CSF sample reflects the composition of biomarkers locally, in the lumbar dural sac. In contrast, analyses of larger volumes may reflect compounds in the rostral spinal or even ventricular CSF, making it essential to standardize future CSF sampling. This is in line with the Consensus Guidelines for CSF and Blood Biobanking.^44^ Several studies have shown negative correlations between CSF monoamine metabolite concentrations and body hight.^41^ It has been proposed that taller persons exhibit a larger surface area for the diffusion of metabolites from the CSF into the surrounding tissues due to a more extended spinal compartment.^46,47^ This is in line with Nilsson et al,^41^ who showed a negative correlation between back length and CSF KYNA levels. However, the present study could not confirm any correlation between back length and any kynurenine pathway metabolites in the CSF.

Previous studies have shown an age-related increase in endogenous KYNA in the rat brain,^48,49^ and CSF of male patients with schizophrenia and bipolar disorder.^50-53^ The age-related increase in central levels of KYNA shown by Kepplinger et al,^54^ has the strength of a broader age range (range 25-74 years), but on the other hand, only patients with acute headaches were investigated which may blur the picture. Sorgdrager et al,^55^ shows an age-dependent increase of KYN, KYNA, and QUIN in serum and CSF. They also established associations between serum and CSF levels of KYN, 3-HK, KYNA, QUIN, and xanthurenic acid; however, their population was older (mean age, 71 years) and was examined after various peripheral neurological conditions. Lumbar punctures in women, initially suspected with meningitis, showed no correlation between KYNA and age, but a positive age-dependent increase in QUIN and negative age correlation with TRP and 3-HK.^56^ However, in the present study, we observed no such age-related increase, which is in line with our previous studies on healthy controls.^41^ This study has the strength of having healthy volunteers, but a limitation is a narrow age span (18-51 years), including only males.

Heavy coffee drinkers show elevated peripheral levels of IL-6,^57^ and IL-6 can induce KYNA production.^58^ Thus, coffee, similar to nicotine,^59^ may influence brain KYNA levels, indicating a confounder in the present data. The present results show the influence of coffee consumption in elevating the CSF TRP fraction 0 to 6 ml, and smoking was associated with lower CSF KYNA in fractions 7 to 12 ml and 13 to 18 ml. Due to the skewness in the groups of coffee drinkers (n = 20) and smokers (n = 3) among subjects, the present results may need further exploration.

Some limitations of our study warrant considerations. Although the mean values of TRP metabolites in brain and blood may not have been greatly affected by the relatively limited number of subjects it may have potentially impacted our ability to explore comprehensive correlations between TRP metabolites across the various compartments studied. Second, while KYNA was the sole metabolite analyzed at different time points throughout the day, investigating additional TRP metabolites over these time intervals could have revealed further correlations. A third limitation is that KYNA was the only TRP metabolite analyzed in urine.

In conclusion, our study provides a comprehensive view of the distribution of TRP metabolites across various body compartments, offering crucial insights for future research on these metabolites in human studies. Overall, our current findings concerning the levels of kynurenines in CSF among healthy individuals are in agreement with previous studies and starkly differs from those seen in patients with infectious diseases where these levels are notably heightened.^60-62^ This incongruity highlights the profound influence of the immune system in modulating the activity of the kynurenine pathway. One significant discovery is the inability to predict brain KYNA levels based solely on plasma analysis. Additionally, our research underscores the necessity of accounting for cerebrospinal fluid (CSF) gradients in planning analyses of the kynurenine pathway within the CSF. Given the observed deviation in TRP metabolites levels in various neurological and psychiatric disorders, our findings could offer a valuable framework for monitoring these metabolites throughout the progression of such illnesses.
